# Supplementary material for: HIVprotI: an integrated web based platform for prediction and design of HIV proteins inhibitors
Source: J Cheminform. 2018 Mar 9;10:12. doi: 10.1186/s13321-018-0266-y (PMC5845081; doi:10.1186/s13321-018-0266-y)
Supplement: Supplementary file 2 — Additional file 2. Source code of HIVProtI web server. [file 13321_2018_266_MOESM2_ESM.zip › HIVprotI_Source-code/hivprotI/weka-3-6-6/doc/weka/attributeSelection/PrincipalComponents.html]

PrincipalComponents


---


|  |  |  |
| --- | --- | --- |
| **Overview** \| **Package** \| **Class** \| **Tree** \| **Deprecated** \| **Index** \| **Help** \| **Weka's home** | |  |
| **PREV CLASS**   **NEXT CLASS** | **FRAMES**    **NO FRAMES**     **All Classes** |  |
| SUMMARY: NESTED \| FIELD \| CONSTR \| METHOD | DETAIL: FIELD \| CONSTR \| METHOD |  |


---


## weka.attributeSelection Class PrincipalComponents

```
java.lang.Object
  weka.attributeSelection.ASEvaluation
      weka.attributeSelection.UnsupervisedAttributeEvaluator
          weka.attributeSelection.PrincipalComponents
```

**All Implemented Interfaces:**: java.io.Serializable, AttributeEvaluator, AttributeTransformer, CapabilitiesHandler, OptionHandler, RevisionHandler

---

``` public class PrincipalComponents extends UnsupervisedAttributeEvaluator implements AttributeTransformer, OptionHandler ```

Performs a principal components analysis and transformation of the data. Use in conjunction with a Ranker search. Dimensionality reduction is accomplished by choosing enough eigenvectors to account for some percentage of the variance in the original data---default 0.95 (95%). Attribute noise can be filtered by transforming to the PC space, eliminating some of the worst eigenvectors, and then transforming back to the original space.


Valid options are:

```
 -D
  Don't normalize input data.
```

```
 -R
  Retain enough PC attributes to account 
  for this proportion of variance in the original data.
  (default = 0.95)
```

```
 -O
  Transform through the PC space and 
  back to the original space.
```

```
 -A
  Maximum number of attributes to include in 
  transformed attribute names. (-1 = include all)
```

**Version:**
:   $Revision: 6690 $

**Author:**
:   Mark Hall (mhall@cs.waikato.ac.nz), Gabi Schmidberger (gabi@cs.waikato.ac.nz)

**See Also:**: Serialized Form

---

**Constructor Summary**

`PrincipalComponents()`


| **Method Summary** | |
| --- | --- |
| `void` | `buildEvaluator(Instances data)`             Initializes principal components and performs the analysis |
| `java.lang.String` | `centerDataTipText()`             Returns the tip text for this property |
| `Instance` | `convertInstance(Instance instance)`             Transform an instance in original (unormalized) format. |
| `double` | `evaluateAttribute(int att)`             Evaluates the merit of a transformed attribute. |
| `Capabilities` | `getCapabilities()`             Returns the capabilities of this evaluator. |
| `boolean` | `getCenterData()`             Get whether to center (rather than standardize) the data. |
| `int` | `getMaximumAttributeNames()`             Gets maximum number of attributes to include in transformed attribute names. |
| `java.lang.String[]` | `getOptions()`             Gets the current settings of PrincipalComponents |
| `java.lang.String` | `getRevision()`             Returns the revision string. |
| `boolean` | `getTransformBackToOriginal()`             Gets whether the data is to be transformed back to the original space. |
| `double` | `getVarianceCovered()`             Gets the proportion of total variance to account for when retaining principal components |
| `java.lang.String` | `globalInfo()`             Returns a string describing this attribute transformer |
| `java.util.Enumeration` | `listOptions()`             Returns an enumeration describing the available options. |
| `static void` | `main(java.lang.String[] argv)`             Main method for testing this class |
| `java.lang.String` | `maximumAttributeNamesTipText()`             Returns the tip text for this property |
| `void` | `setCenterData(boolean center)`             Set whether to center (rather than standardize) the data. |
| `void` | `setMaximumAttributeNames(int m)`             Sets maximum number of attributes to include in transformed attribute names. |
| `void` | `setOptions(java.lang.String[] options)`             Parses a given list of options. |
| `void` | `setTransformBackToOriginal(boolean b)`             Sets whether the data should be transformed back to the original space |
| `void` | `setVarianceCovered(double vc)`             Sets the amount of variance to account for when retaining principal components |
| `java.lang.String` | `toString()`             Returns a description of this attribute transformer |
| `java.lang.String` | `transformBackToOriginalTipText()`             Returns the tip text for this property |
| `Instances` | `transformedData(Instances data)`             Gets the transformed training data. |
| `Instances` | `transformedHeader()`             Returns just the header for the transformed data (ie. |
| `java.lang.String` | `varianceCoveredTipText()`             Returns the tip text for this property |

**Methods inherited from class weka.attributeSelection.ASEvaluation**

`forName, makeCopies, postProcess`

**Methods inherited from class java.lang.Object**

`equals, getClass, hashCode, notify, notifyAll, wait, wait, wait`

**Constructor Detail**

### PrincipalComponents

```
public PrincipalComponents()
```


**Method Detail**

### globalInfo

```
public java.lang.String globalInfo()
```

:   Returns a string describing this attribute transformer

    :   **Returns:**: a description of the evaluator suitable for displaying in the explorer/experimenter gui

---


### listOptions

```
public java.util.Enumeration listOptions()
```

:   Returns an enumeration describing the available options.

    :   **Specified by:**: `listOptions` in interface `OptionHandler`
    :   **Returns:**: an enumeration of all the available options.

---


### setOptions

```
public void setOptions(java.lang.String[] options)
                throws java.lang.Exception
```

:   Parses a given list of options.


    Valid options are:

    ```
     -D
      Don't normalize input data.
    ```

    ```
     -R
      Retain enough PC attributes to account 
      for this proportion of variance in the original data.
      (default = 0.95)
    ```

    ```
     -O
      Transform through the PC space and 
      back to the original space.
    ```

    ```
     -A
      Maximum number of attributes to include in 
      transformed attribute names. (-1 = include all)
    ```

    :   **Specified by:**: `setOptions` in interface `OptionHandler`
    :   **Parameters:**: `options` - the list of options as an array of strings **Throws:**: `java.lang.Exception` - if an option is not supported

---


### centerDataTipText

```
public java.lang.String centerDataTipText()
```

:   Returns the tip text for this property

    :   **Returns:**: tip text for this property suitable for displaying in the explorer/experimenter gui

---


### setCenterData

```
public void setCenterData(boolean center)
```

:   Set whether to center (rather than standardize)
    the data. If set to true then PCA is computed
    from the covariance rather than correlation matrix.

    :   **Parameters:**: `center` - true if the data is to be centered rather than standardized

---


### getCenterData

```
public boolean getCenterData()
```

:   Get whether to center (rather than standardize)
    the data. If true then PCA is computed
    from the covariance rather than correlation matrix.

    :   **Returns:**: true if the data is to be centered rather than standardized.

---


### varianceCoveredTipText

```
public java.lang.String varianceCoveredTipText()
```

:   Returns the tip text for this property

    :   **Returns:**: tip text for this property suitable for displaying in the explorer/experimenter gui

---


### setVarianceCovered

```
public void setVarianceCovered(double vc)
```

:   Sets the amount of variance to account for when retaining
    principal components

    :   **Parameters:**: `vc` - the proportion of total variance to account for

---


### getVarianceCovered

```
public double getVarianceCovered()
```

:   Gets the proportion of total variance to account for when
    retaining principal components

    :   **Returns:**: the proportion of variance to account for

---


### maximumAttributeNamesTipText

```
public java.lang.String maximumAttributeNamesTipText()
```

:   Returns the tip text for this property

    :   **Returns:**: tip text for this property suitable for displaying in the explorer/experimenter gui

---


### setMaximumAttributeNames

```
public void setMaximumAttributeNames(int m)
```

:   Sets maximum number of attributes to include in
    transformed attribute names.

    :   **Parameters:**: `m` - the maximum number of attributes

---


### getMaximumAttributeNames

```
public int getMaximumAttributeNames()
```

:   Gets maximum number of attributes to include in
    transformed attribute names.

    :   **Returns:**: the maximum number of attributes

---


### transformBackToOriginalTipText

```
public java.lang.String transformBackToOriginalTipText()
```

:   Returns the tip text for this property

    :   **Returns:**: tip text for this property suitable for displaying in the explorer/experimenter gui

---


### setTransformBackToOriginal

```
public void setTransformBackToOriginal(boolean b)
```

:   Sets whether the data should be transformed back to the original
    space

    :   **Parameters:**: `b` - true if the data should be transformed back to the original space

---


### getTransformBackToOriginal

```
public boolean getTransformBackToOriginal()
```

:   Gets whether the data is to be transformed back to the original
    space.

    :   **Returns:**: true if the data is to be transformed back to the original space

---


### getOptions

```
public java.lang.String[] getOptions()
```

:   Gets the current settings of PrincipalComponents

    :   **Specified by:**: `getOptions` in interface `OptionHandler`
    :   **Returns:**: an array of strings suitable for passing to setOptions()

---


### getCapabilities

```
public Capabilities getCapabilities()
```

:   Returns the capabilities of this evaluator.

    :   **Specified by:**: `getCapabilities` in interface `CapabilitiesHandler` **Overrides:**: `getCapabilities` in class `ASEvaluation`
    :   **Returns:**: the capabilities of this evaluator **See Also:**: `Capabilities`

---


### buildEvaluator

```
public void buildEvaluator(Instances data)
                    throws java.lang.Exception
```

:   Initializes principal components and performs the analysis

    :   **Specified by:**: `buildEvaluator` in class `ASEvaluation`
    :   **Parameters:**: `data` - the instances to analyse/transform **Throws:**: `java.lang.Exception` - if analysis fails

---


### transformedHeader

```
public Instances transformedHeader()
                            throws java.lang.Exception
```

:   Returns just the header for the transformed data (ie. an empty
    set of instances. This is so that AttributeSelection can
    determine the structure of the transformed data without actually
    having to get all the transformed data through transformedData().

    :   **Specified by:**: `transformedHeader` in interface `AttributeTransformer`
    :   **Returns:**: the header of the transformed data. **Throws:**: `java.lang.Exception` - if the header of the transformed data can't be determined.

---


### transformedData

```
public Instances transformedData(Instances data)
                          throws java.lang.Exception
```

:   Gets the transformed training data.

    :   **Specified by:**: `transformedData` in interface `AttributeTransformer`
    :   **Returns:**: the transformed training data **Throws:**: `java.lang.Exception` - if transformed data can't be returned

---


### evaluateAttribute

```
public double evaluateAttribute(int att)
                         throws java.lang.Exception
```

:   Evaluates the merit of a transformed attribute. This is defined
    to be 1 minus the cumulative variance explained. Merit can't
    be meaningfully evaluated if the data is to be transformed back
    to the original space.

    :   **Specified by:**: `evaluateAttribute` in interface `AttributeEvaluator`
    :   **Parameters:**: `att` - the attribute to be evaluated **Returns:**: the merit of a transformed attribute **Throws:**: `java.lang.Exception` - if attribute can't be evaluated

---


### toString

```
public java.lang.String toString()
```

:   Returns a description of this attribute transformer

    :   **Overrides:**: `toString` in class `java.lang.Object`
    :   **Returns:**: a String describing this attribute transformer

---


### convertInstance

```
public Instance convertInstance(Instance instance)
                         throws java.lang.Exception
```

:   Transform an instance in original (unormalized) format. Convert back
    to the original space if requested.

    :   **Specified by:**: `convertInstance` in interface `AttributeTransformer`
    :   **Parameters:**: `instance` - an instance in the original (unormalized) format **Returns:**: a transformed instance **Throws:**: `java.lang.Exception` - if instance cant be transformed

---


### getRevision

```
public java.lang.String getRevision()
```

:   Returns the revision string.

    :   **Specified by:**: `getRevision` in interface `RevisionHandler` **Overrides:**: `getRevision` in class `ASEvaluation`
    :   **Returns:**: the revision

---


### main

```
public static void main(java.lang.String[] argv)
```

:   Main method for testing this class

    :   **Parameters:**: `argv` - should contain the command line arguments to the evaluator/transformer (see AttributeSelection)


---


|  |  |  |
| --- | --- | --- |
| **Overview** \| **Package** \| **Class** \| **Tree** \| **Deprecated** \| **Index** \| **Help** \| **Weka's home** | |  |
| **PREV CLASS**   **NEXT CLASS** | **FRAMES**    **NO FRAMES**     **All Classes** |  |
| SUMMARY: NESTED \| FIELD \| CONSTR \| METHOD | DETAIL: FIELD \| CONSTR \| METHOD |  |


---
